# Preoperative Periapical Status and Outcomes of Vital Pulp Therapy: A Systematic Review and Meta‐Analysis

**DOI:** 10.1111/aej.70042

**Published:** 2025-11-30

**Authors:** Isadora de Souza Basso, Laura Huffel Dotto, Patrícia Maria Poli Kopper, Roberta Kochenborger Scarparo

**Affiliations:** ^1^ Graduate Program in Dentistry Federal University of Rio Grande do Sul (UFRGS) Porto Alegre Brazil

**Keywords:** case selection, endodontics, periapical status, pulpotomy, vital pulp therapy

## Abstract

This systematic review has investigated whether the preoperative periapical radiographic status (PPRS) is a predictor of VPT success/survival. Four databases were searched for studies published from inception to November 2024. The studies' characteristics were tabulated, and a meta‐analysis was performed to evaluate the correlation between PPRS and the success rate of VPT. The QUIPS tool and the GRADE criteria were used to assess the risk of bias and the certainty of evidence. No significant association between preoperative periapical radiolucency and full pulpotomy success was observed (OR 0.75, 95% CI 0.30–1.87; *p* = 0.54; Z = 0.61), though heterogeneity among studies was noted (*Τ* ^2^ = 0.41; *χ* ^2^ = 0.29; *I* ^2^ = 59%). Overall high risk of bias was detected. The certainty of evidence was rated as low. The findings suggest that periapical radiolucency should not contraindicate VPT. However, the certainty of evidence regarding this prognostic factor should be improved to refine diagnostic criteria and guide clinical decision‐making.

## 1 Introduction

There has been ongoing debate about whether vital pulp therapies could be considered definitive treatments for mature permanent teeth. In this regard, pulp diagnosis has been reframed to contemplate the evaluation of the dental pulp's ability to heal. For this purpose, the identification of prognostic factors for vital pulp therapy (VPT) is one of the significant issues that should be further studied [1], and VPT should be indicated since pulp vitality and its capacity to recover post‐treatment are presumed.

Pulp diagnosis is crucial for assertive clinical decisions, but the current methods of ascertaining healing ability are subjective [1]. The diagnosis is usually based on symptoms, clinical history, sensibility testing, and other findings from clinical and radiographic analysis. Molecular diagnostic tests are not yet available. Besides dental pulp vitality, the tissue consistency, as well as its bleeding intensity, coloration, and time for haemostasis, are considered for VPT indication. Within this context, periapical status, as observed in imaging exams, has also been considered for treatment choices [1, 2, 3].

Periodontal ligament widening and periapical radiolucency are commonly associated with pulp necrosis and apical periodontitis [4, 5], and most of the studies that evaluate VPT outcomes exclude teeth presenting this radiographic periapical status [6, 7, 8]. However, radiographic examination alone cannot detect pulpal inflammation, and other factors must be analysed to confirm apical periodontitis [1, 9, 10]. Periodontal ligament widening and periapical radiolucency can respond positively to sensibility tests have been described due to vascular supply [11, 12]. A possible explanation should be that, during pulp inflammation, interleukins (IL‐1, IL‐6), tumoral growth factor‐alpha (TNF‐α), and prostaglandin (PGE2) are overexpressed, thus eliciting a periapical response [13]. It is uncertain whether preoperative periapical radiolucency in vital teeth would affect VPT outcomes.

While an observational study reported high success rates of pulpotomies performed in teeth presenting both preoperative periodontal ligament widening and periapical radiolucency and responding positively to pulp sensibility tests [14], other authors point out insufficient evidence to confirm the total regression of periapical lesions following VPT [15]. Considering these divergencies, the influence of preoperative periapical radiolucency on VPT success is still to be elucidated. This systematic review and meta‐analysis have compiled data on the association between preoperative radiolucency and VPT clinical and radiographic success.

## 2 Material and Methods

### 2.1 Protocol Registration

This systematic review was performed according to the Checklist for Critical Appraisal and data extraction for systematic reviews of prediction Modelling Studies CHARMS checklist [16], and reported according to the Preferred Reporting Items for Systematic Reviews and Meta‐Analyses (PRISMA) statement [17]. A protocol based on the PRISMA 2020 statement was previously registered at the International Prospective Register of Systematic Reviews (PROSPERO) [18] under the code CRD4203463496. An amendment was submitted for data analysis settings.

### 2.2 Research Question and Eligibility Criteria

- The research question was formulated using the acronym PICOTS [19, 20];
  ○ (P) Participants with permanent teeth undergoing VPT;
  ○ (I) periapical radiographic status on the day of treatment;
  ○ (C) gender, age, systemic condition, preoperative symptoms/diagnosis, caries extent/location, restoration extent/type, bleeding time;
  ○ (O) clinical and radiographic success and survival;
  ○ (T) data collected immediately before the clinical procedure and follow‐up performed at least one year later;
  ○ (S) Clinical trials and observational studies developed in dental schools, and private and public dental services.

Clinical studies (cohort studies and clinical trials) were eligible if they evaluated the clinical/radiographic success and/or survival rates of VPT in permanent teeth with exposed dental pulps (direct pulp capping, partial pulpotomy or full pulpotomy). The mean follow‐up time was at least one year, and the effect of periapical radiographic status on VPT success/survival outcome could be collected. Calcium hydroxide (pro‐analysis powder or paste formulations), MTA, or other bioceramic materials should be used as the pulp capping material. No language restrictions were applied. Exclusion criteria were case reports and case series; studies for which a full text could not be obtained after search in journals, interinstitutional commuting, and author contact; and studies that did not include definitive tooth restoration in the clinical protocols.

### 2.3 Information Sources and Search Strategy

Electronic searches were conducted by two independent authors (IB and LD) in the following electronic databases: PubMed MEDLINE (https://pubmed.ncbi.nlm.nih.gov/), Latin American and Caribbean Health Sciences Literature (LILACS) (https://lilacs.bvsalud.org/en), EMBASE (https://embase.com) and Web of Science (https://access.clarivate.com). The search used a combination of the following: (‘Pulpotomy’ OR ‘Pulpotomies’) OR (‘Dental Pulp Capping’ OR ‘Pulp Capping, Dental’ OR ‘Pulp Capping’ OR ‘Capping, Pulp’ OR ‘Cappings, Pulp’ OR ‘Pulp Cappings’ OR ‘Capping, Dental Pulp’ OR ‘Cappings, Dental Pulp’ OR ‘Dental Pulp Cappings’ OR ‘Pulp Cappings, Dental’) AND (‘Treatment outcome’ OR ‘Outcome, Treatment’ OR ‘Clinical Efficacy’ OR ‘Efficacy, Clinical’). Search strategies were adapted for each database (Table 1). No filters, limits, or publication date restrictions were applied. All searches were conducted from the earliest date available until November 2024. The references were managed using reference software (EndNote X7; Thomson Reuters, Philadelphia, PA), in which reference collection and duplicate removal were carried out.

**TABLE 1:** Search strategy used and results for each electronic database.

Database	Query
PubMed	(‘Pulpotomy’ OR ‘Pulpotomies’) OR (‘Dental Pulp Capping’ OR ‘Pulp Capping, Dental’ OR ‘Pulp Capping’ OR ‘Capping, Pulp’ OR ‘Cappings, Pulp’ OR ‘Pulp Cappings’ OR ‘Capping, Dental Pulp’ OR ‘Cappings, Dental Pulp’ OR ‘Dental Pulp Cappings’ OR ‘Pulp Cappings, Dental’) AND (‘Treatment outcome’ OR ‘Outcome, Treatment’ OR ‘Clinical Efficacy’ OR ‘Efficacy, Clinical’)
Embase	(‘pulpotomy’/exp. OR pulpotomy OR ‘tooth’/exp. OR tooth) AND (‘pulpotomy’/exp. OR pulpotomy OR ‘tooth’/exp. OR tooth) AND (‘pulpotomy’/exp. OR pulpotomy) OR (‘dental’/exp. OR dental) AND (‘pulp’/exp. OR pulp) AND (‘capping’/exp. OR capping) OR (‘dental’/exp. OR dental) AND (‘pulp’/exp. OR pulp) AND (‘capping’/exp. OR capping) OR (‘pulp’/exp. OR pulp) AND (‘cap’/exp. OR cap) AND (‘procedure’/exp. OR procedure) OR (‘pulp’/exp. OR pulp) AND (‘cap’/exp. OR cap) AND (‘procedure’/exp. OR procedure) OR (‘pulp’/exp. OR pulp) AND (‘cap’/exp. OR cap) AND (‘technique’/exp. OR technique) OR (‘pulp’/exp. OR pulp) AND (‘cap’/exp. OR cap) AND (‘technique’/exp. OR technique) OR (‘vital’/exp. OR vital) AND (‘pulp’/exp. OR pulp) AND (‘therapy’/exp. OR therapy) OR (‘vital’/exp. OR vital) AND (‘pulp’/exp. OR pulp) AND (‘therapy’/exp. OR therapy) AND ((‘treatment’/exp. OR treatment) AND (‘outcome’/exp. OR outcome) OR ‘treatment’/exp. OR treatment) AND (‘outcome’/exp. OR outcome) OR (‘clinical’/exp. OR clinical) AND (‘outcome’/exp. OR outcome) OR (‘clinical’/exp. OR clinical) AND (‘outcome’/exp. OR outcome) OR (‘clinical’/exp. OR clinical) AND (‘treatment’/exp. OR treatment) AND (‘outcome’/exp. OR outcome) OR (‘clinical’/exp. OR clinical) AND (‘treatment’/exp. OR treatment) AND (‘outcome’/exp. OR outcome) OR (‘therapy’/exp. OR therapy) AND (‘outcome’/exp. OR outcome) OR (‘therapy’/exp. OR therapy) AND (‘outcome’/exp. OR outcome) OR (‘therapy’/exp. OR therapy) AND (‘efficacy’/exp. OR efficacy)
Lilacs	((pulpotomy) OR (pulpotomies) OR (pulpotomia) OR (pulpotomía) OR (dental pulp capping) OR (capeamento da polpa dentária) OR (recubrimiento dela pulpa dental)) AND ((treatment outcome) OR (resultado do tratamento) OR (resultado del tratamiento) OR (treatment efficacy) OR (eficácia do tratamento) OR (eficacia del tratamiento))
Web of Science	(ALL = ((Pulpotomy) OR (pulpotomies) OR (dental pulp capping) OR (pulp capping) OR (capping, pulp) OR (cappings, pulp) OR (pulp cappings) OR (capping, dental pulp) OR (cappings, dental pulp) OR (dental pulp cappings))) AND (ALL = ((treatment outcome) OR (outcome, treatment) OR (clinical efficacy) OR (efficacy, clinical)))

### 2.4 Data Collection Process, Data Items, and Effect Measures

Two reviewers (IB and LD) separately applied the eligibility criteria during the title, abstract, and full‐text screening. In cases of disagreement at any stage of the search, the reviewers met for discussion, and two senior investigators (PK and RS) defined a consensus.

**FIGURE 1:**
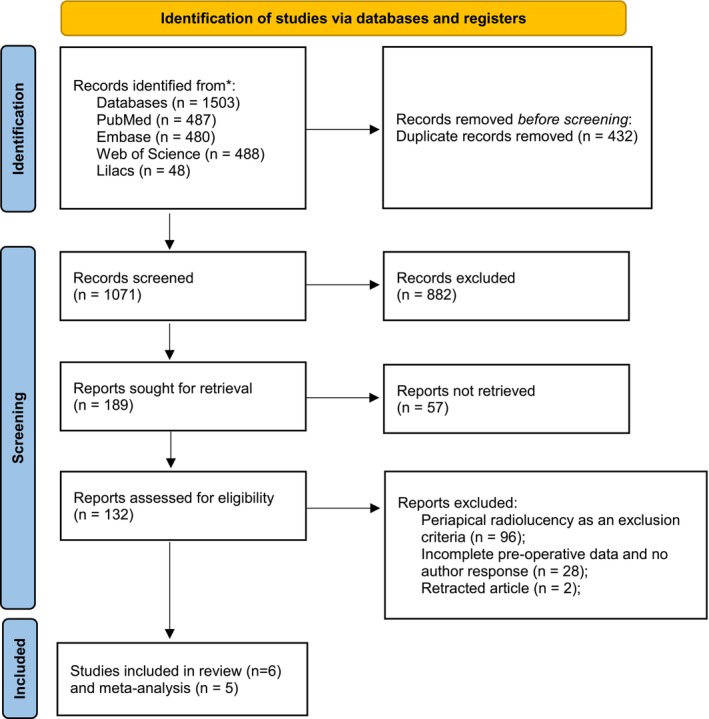
Prisma 2020 flow diagram for new systematic reviews which included searches of databases and registers only.

After the selection process, the reference lists of included studies and reviews were manually checked to identify potentially relevant articles. New studies were also searched in the references of the included studies. The PRISMA flow chart (Figure 1) illustrates the selection process.

The same two authors (IB and LD) extracted data separately into a standardised data spreadsheet in Microsoft Office Excel 2016 (Microsoft Corporation, Redmond, WA). Data extracted included first author's names, year of publication, participants' recruitment criteria, place of treatment, treatment provider, VPT modality (direct pulp capping, partial pulpotomy or full pulpotomy), study design, sample size (number of treated teeth and teeth followed up), type of pulp exposure, preoperative condition, self‐reported participants' age on the day of treatment (range, mean/median), capping material, the pulpal haemostasis method, type of coronal sealing, follow‐up time, overall success rates and success rates according to outcomes of statistical analysis, performed to assess the associations between periapical radiographic status and treatment outcomes, and covariates included the multivariate prediction models (if available) (Table 2). The classification of treatment outcomes followed the authors' criteria, which usually considered clinical and radiographic evaluation based on the AAE or ESE recommendations [10, 26].

**TABLE 2:** Characteristics of the studies included in the qualitative analysis (*n* = 6).

First author/year	Study design	Number of teeth: Attended follow up/Treated	Participants Recruitment/Treatment provider	Type of pulp exposure	Preoperative condition	Age (years‐old)	Capping material	Irrigant for wound surface cleaning	Coronal sealing	Follow‐ up time	Sucess rate % (successful sample/total sample)	Statistical outcomes described for the association of age × treatment outcome	
												Univariate/Bivariate analysis	Multivariate analysis (covariates included)
*Full pulpotomy*													
Asgary 2014 [21]	Randomised Clinical Trial	137/205	Consecutively treated patients from health care centers of medical Universities/NI	Carious	SIP	*Range*: 9–65 *Mean*: 35.5	CEM	Sterile saline	Amalgam	1.5 years *Range*; *Mean*/*Median*: NI	*Overall*: 78.1 *According to PS*: (−) = 82.4 (84/102) (+) = 65.7 (23/35)	Multiple Binary logistic regression model *p* = 0.71	Not performed
Qudeimat 2016 [22]	Prospective cohort study	23/23	Eligible participants recruited from Dental School/one single endodontist	Carious lesions	SIP	*Range*: 7.6–13.6	*Mean*: 10.7 ± 1.7	White and Grey MTA	N 5% NaOCl	Glass–ionomer filling and stainless‐steel crown	*Range*: 18.9–73.6 months *Mean*: 57.5 ± 13.9 months	*Overall*: 100.0 *According to PS*: (−) = 100 (19/19) (+) = 100 (4/4)	Statistical analysis not performed to evaluate the association between periapical status and treatment success
Asgary 2022 [23]	Randomised Clinical Trial	98/106	Consecutively treated patients from Dental School/Graduate students	Carious	AP, SIP	*Range*: 16–60 *Mean*: 38	ProRoot MTA CEM	0.2% Chlorhexidine, sterile saline, 5.25% NaOCl	Glass ionomer and light‐cured resin‐bonded dental composite restorations	2 years *Range*; NI *Mean*: 26.98 ± 4.91 (MTA) and 27.83 ± 5.03 months (CEM)	*Overall*: MTA:100 (51/51) CEM: 97.9 (46/47) *According to PS*: (−) =100 (64/64) (+) = 97 (33/34)	Statistical analysis not performed to evaluate the association between periapical status and treatment success	
Elmas 2023 [15]	Prospective cohort study	40/50	Eligible participants recruited from Dental School/one single endodontist	Carious lesions	SIP	*Range*: 6–14 *Mean*: 10 *Median*: 10	ProRoot White MTA	Saline solution	Composite restoration or stainless‐steel crown	*Range*: 12–24 months *Mean/Median*: 18 months	*Overall*: 1 year: 98 (49/50) 2 years: 97.5 (39/40) *According to PS*: (−) = 100 (17/17) (+) = 97.9 (32/33)	Statistical analysis not performed to evaluate the association between periapical status and treatment success	
Asgary 2024 [24]	Retrospective Cohort Study	378/1149	Consecutively treated patients from Private Dental Clinic/Single endodontist	Carious	AP, SIP	Range: NI *Mean*: 41.27 ± 11.63 (females) 41.59 ± 10.74 (males)	CEM	Sterile saline and 5.25% NaOCl	Silver amalgam or composite resin	*Range*: *Mean*: 42.21 months Median: 39 months	*Overall*: 91.6 *According to PS*: (−) = 89.9 (204/227) (+) = 87.4 (132/151)	Cox proportional hazards model analyses *p* > 0.5 Full Pulpotomy^a^ HR 1.19; CI (0.839–4.388); *p* = 0.122	Multivariate Cox proportional hazards model analyses Full Pulpotomy^a^ HR 2.105; CI (0.847–5.234) *p* = 0.109 (Symptoms, radiographic signs of apical periodontitis, restoration type, restoration surfaces, age, gender, tooth type, time for haemostasis, presence of preoperative restoration)
*Partial and full pulpotomy*													
Taha 2024 [25]	Randomised Clinical Trial	189/200	Eligible participants recruited from Dental School/Single graduate student	Deep caries or extremely deep caries	SIP	*Range*: 11–61 *Mean*: 27.76 ± 11.69	Neo PUTTY	2 5% NaOCl	Composite Resin	6 months and 1 year *Range/Mean/Median*: 9 months	*Overall*: 94.5 *According to PS* ^b^: (−) = 89.3 (125/140) (+) = 100 (60/60)	Statistical analysis not performed to evaluate the association between periapical status and treatment success	

Abbreviations: (−), cases for which no periapical widening or radiolucency was described; (+), cases for which periapical widening or radiolucency was described; AP, apical periodontitis; CEM, calcium‐enriched mixture; MTA, mineral trioxide aggregate; NI, not informed; PS, periapical status; SIP, symptomatic irreversible pulpitis.

^a^ HR calculated based on data provided by authors after request.

^b^ Information based on the total number of treated teeth since there's no additional information of preoperative condition in the escaped cases.

To ensure data saturation, corresponding authors of the included studies were contacted by e‐mail requesting more information when the data described above were not reported in the manuscript. An attempt was made to contact, and the wait time for a response was 10 days.

### 2.5 Data Analysis

A meta‐analysis was performed to assess the prognostic value of periapical status in teeth that underwent full pulpotomy. The RevMan software (version 5.4—The Cochrane Collaboration, Denmark) was used for analyses. Only a meta‐analysis based on the bivariate estimated effect could be performed. Data about sample size and successful events in teeth with periodontal ligament widening/periapical radiolucency were pooled and compared to that from teeth showing no radiographic alteration in the periapical region. Analyses were performed using the Mantel–Haenszel method, a random effects model, estimating odds ratio (OR) based on the total number of participants and successful events in each periapical status category, with a 95% confidence interval (95% CI). Heterogeneity was assessed using *Τ* ^2^, *I* ^2^, and *χ* ^2^ statistics.

### 2.6 Risk of Bias and Applicability Assessment

Two independent reviewers (IB, LD), previously calibrated by discussing the checklist items, assessed the quality of included studies using the QUality In Prognostic factor Studies (QUIPS) checklist [27]. Any disagreements were resolved by discussion between the reviewers and other team members (PK, RS).

QUIPS analysed six domains: (1) bias due to study participation, (2) bias due to attrition, (3) bias due to prognostic factor measurement, (4) bias due to outcome measurement, (5) bias due to confounding, and (6) bias in statistical analysis and reporting. Publication bias was assessed using Begg's test.

### 2.7 Certainty of Evidence Assessment

Certainty of evidence and strength of recommendation were assessed using the Grading of Recommendations, Development, and Evaluation (GRADE) approach [25, 28, 29] for the domains ‘risk of bias’, ‘indirectness’, ‘inconsistency’, ‘imprecision’, and ‘publication bias’. The assessment was carried out by two independent reviewers (IB and LD), and disagreements were resolved by a senior investigator (RS), who considered the adaptations suggested for prognostic reviews. The GRADEpro Guideline Development Tool (GRADEpro GDT, 2022) was used, and evidence was rated as ‘very low’, ‘low’, ‘moderate’, and ‘high’.

## 3 Results

### 3.1 Studies Selection and Characteristics

The search strategy of this review resulted in 1503 studies: 487 from PubMed, 48 from LILACS, 480 from EMBASE, and 488 from Web of Science (Table 1, Figure 1). After discarding 432 duplicates, 1071 studies were eligible for title analysis, and 189 for abstract screening. One hundred and thirty‐two reports were assessed for eligibility; in total, 128 studies were excluded for the following reasons: studies in which periapical radiolucency was an exclusion criterion (*n* = 96); studies with incomplete pre‐operative information and without author response (*n* = 28); retracted article (*n* = 2) (Table 1). Six studies fulfilled the eligibility criteria and were qualitatively described. Full pulpotomy was the only VPT modality observed in 5 studies, and one described the pooled results of full and partial pulpotomy [21]. Four studies included only cases presenting irreversible symptomatic pulpitis, and the other two also considered asymptomatic teeth and cases diagnosed as reversible pulpitis. The study's participants' ages ranged from 9 to 65 years old. Dental caries was the aetiology of pulp disease in all studies. The treatment providers varied among the studies and included graduate students and specialists. Sodium hypochlorite, saline solution, and chlorhexidine were used in the study protocols. MTA and CEM were the most used capping materials (Table 2).

VPT showed an overall success rate from 78.1% to 100% of the treated teeth. Bivariate statistical analysis for assessing the association between preoperative periapical status and VPT success was performed in only one study [22]; in three studies, the bivariate association was calculated based on the manuscript's descriptive data [15, 23, 24]; in another [30], the bivariate and multivariate association between periapical status and treatment survival was calculated based on additional data provided by the authors after request. One study [21] was not included in the meta‐analysis since full pulpotomy quantitative data were not provided by the authors.

### 3.2 Data Extraction

**FIGURE 2:**
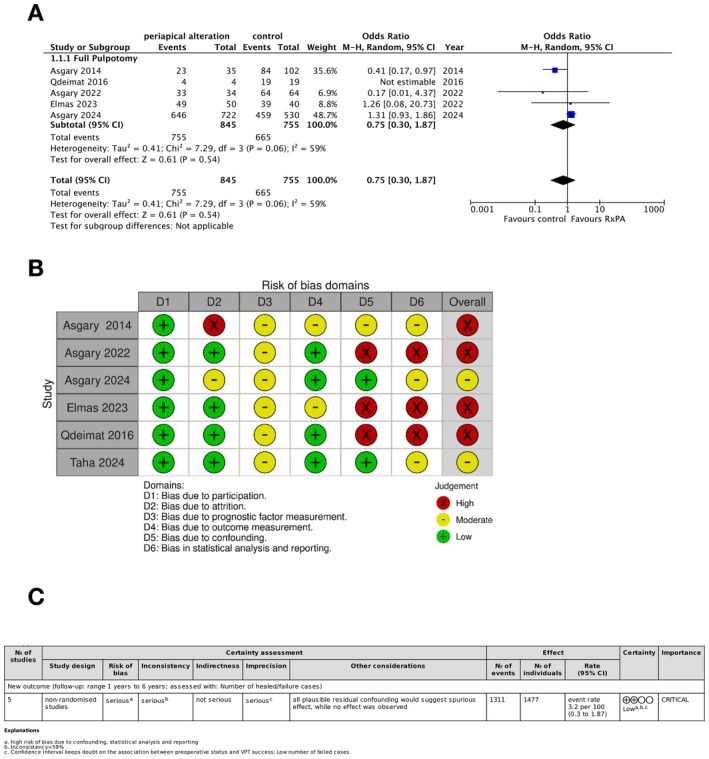
(A) Meta‐analysis of the association between preoperative status and Full Pulpotomy success; (B) Risk of bias of the studies included in qualitative and quantitative analysis according to QUIPS tool domains; (C) Certainty of evidence according to GRADE criteria.

Five out of the six included studies were meta‐analysed, showing no association between preoperative periapical radiographic status and full pulpotomy outcomes (OR 0.75, 95% CI 0.30–1.87; *p* = 0.54; *Z* = 0.61) (Figure 2A). Heterogeneity among the studies was statistically significant (*Τ* ^2^ = 0.41; *χ* ^2^ = 7.29; *I* ^2^ 59%).

### 3.3 Methodological Quality and Risk of Bias Assessment

According to QUIPS tool, out of the 6 studies included in the qualitative analysis, 4 showed high and 2 presented moderate risk of bias (Figure 2B). Five of these studies were included in the quantitative analysis, and the overall high risk of bias was classified as high. Bias related to confounding and statistical analysis, and reporting were the main concerns. Egger's test did not reveal publication bias.

### 3.4 Certainty of Evidence

Certainty of evidence was rated as low. Risk of bias, inconsistency, and imprecision have lowered the strength of recommendation (Figure 2C).

## 4 Discussion

The present study compiled the available scientific data on the association of preoperative periapical status and VPT success, showing no significant outcomes and revealing the need for further clinical investigations to strengthen the certainty of evidence on this prognostic factor.

It has been discussed that periapical inflammatory infiltrates, increased osteoclast numbers, and bone destruction can occur before pulpal necrosis, due to the periapical tissue's response to pulp inflammation, which has been described even in early pulpitis [31, 32, 33]. This understanding underscores the complexity of the relationship between pulpal and periapical pathologies and highlights the importance of thorough clinical evaluation in diagnosing and managing such conditions [32]. Considering the available published data, it is not possible to affirm that alterations in the radiographic periapical status are a reliable predictive measure of the ability of pulp to recover.

A previous narrative review has discussed the factors affecting the outcomes of VPT, suggesting that pre‐, intra‐, and postoperative prognostic factors should be further studied. In this regard, a systematic review evaluating patients' age impact on VPT outcomes has emphasised the relevance of deepening knowledge about clinically measurable prognostic factors to provide scientifically based decision‐making on VPT indication [34].

Periapical status is among the prognostic factors that should be considered. A recent systematic review evaluating prognostic factors of VPT has suggested better full pulpotomy outcomes in teeth showing no pre‐treatment periapical involvement, although the results were based only on one clinical study [35]. In disagreement, a non‐systematic review has discussed this topic, suggesting that VPT can yield positive results in teeth with apical periodontitis when vital tissue is evident after pulp exposure. However, the authors emphasise that it is not clear how predictable VPT is in teeth with apical radiolucency. The current systematic approach is in line with the last conclusions, since it has demonstrated no significant impact of periapical status on full pulpotomy outcomes.

The results presented herein relativize the relevance of preoperative radiographic periapical status as a predictor of VPT outcomes. Among the possible explanations for the present findings, the limitations of image exams must be discussed.

The accuracy of imaging exams evaluating periapical status has been frequently questioned [36, 37]. Radiological diagnosis may not reflect all clinical and biological features, and radiographs may not always accurately represent histologic periapical findings [26, 38, 39]. The definition of where widening ends and radiolucency begins can be subjective, as can the assessment of pulp health and disease states based on imaging exams. Even so, periapical radiographs are still recommended to assess periapical status when deciding on VPT or RCT [10, 26], and the observation of periodontal ligament widening and periapical radiolucency is frequently reported to contraindicate maintaining dental pulp vitality [7]. In this regard, out of the 132 manuscripts available for full‐text review in this study, 126 were not included because considered periapical status as eligibility criteria during sample selection. Alternatively, the inference that preoperative periapical status is a relevant predictor for VPT success has motivated the suggestion of a combination of RCT and VPT for multirooted teeth diagnosed as irreversible symptomatic pulpitis and showing a periapical radiolucency in only one of the roots [40].

On the other hand, some authors demonstrated that contraindicating VPT based only on the preoperative periapical radiographic status could lead to misdiagnosing pulp irreversible diseases (false positives), thus causing overtreatments [22, 23]. This position agrees with the current meta‐analysis results, showing no differences in full pulpotomy success according to preoperative periapical status. In this regard, to avoid possible misdiagnosis based only on the imaging exam [36], the current recommended analysis of conventional radiography should be considered together with other clinical features. Besides, improvement of diagnostic tools for pulp health assessment is needed [41, 42].

One can infer that cone beam computed tomography (CBCT) image exams would help to establish the periapical status more accurately, and thus to more assertive decision making for VPT. However, currently, there is no scientific evidence that proves 3D imaging displays to favour VPT case selection. Measuring problems in marginal bone levels [43] and false positive diagnoses [44] are probably among the concerns that contributed to AAE and ESE guidelines' recommendation of not using CBCT as a routine for the evaluation of dental pulp condition [10, 26].

This review reveals the need for improving data analysis and reporting in clinical studies to avoid confounders and to enable a more comprehensive understanding of prognostic factors. Bias related to confounding, outcome measurements/statistical analysis, and reporting was the main concern in the available evidence.

Since few studies were included in the meta‐analyses, and multivariate analysis could not be performed based on the reported data, the present results should be interpreted with caution.

This study highlights the need for adjustments in the eligibility criteria of clinical studies that evaluate the results of VPT. Based on the current review, using preoperative periapical radiographic status as an exclusion criterion is not justified and impairs a robust evaluation of this candidate predictor of treatment outcome.

Clinical studies should also provide more detailed descriptions of participants' characteristics that could impact case selection, and multivariate regression analysis should be carried out to increase data reliability. In this regard, besides the traditional domains considered in the methodological quality of clinical trials and observational studies [45], specific issues concerning study participation, attrition, prognostic factors, and outcome measurements, confounding, and statistical analysis and reporting should be observed [27, 46, 47].

In the present review, QUIPS (Quality In Prognosis Studies) was chosen for quality analysis, aiming to contemplate the analysis of risk of bias in the context of prognostic factors assessment [27, 46, 47]. QUIPS shows advantages compared to traditional study quality analysis tools, such as ROB2 and ROBINs, for reviews of prognostic factors, since it prioritises concerns related to the analysis of the predictive value of certain issues regardless of the study's design. In this regard, the domains established in this tool should also guide the design and reporting in clinical studies.

Further attention should also be given to the development of reviews with a focus on VPT prognostic factors. To date, only one systematic review of prognostic studies has been performed to evaluate VPT predictors [34], but this approach presents potential to summarise data and help in clinical decisions.

## 5 Conclusion

This systematic review and meta‐analysis showed that preoperative periapical radiolucency is an unreliable predictor of VPT outcome. Despite that, this review suggests that the periapical condition does not necessarily mean that VPT should not be considered. Further analysis in well‐designed clinical studies is required to improve the certainty of evidence related to the association between periapical radiolucency and VPT outcomes.

## Author Contributions

All authors have contributed significantly and agree with the manuscript.

## Funding

The authors have nothing to report.

## Conflicts of Interest

The authors declare no conflicts of interest.

## Data Availability

The data that support the findings of this study are available from the corresponding author upon reasonable request.
